# Bortezomib attenuates renal interstitial fibrosis in kidney transplantation via regulating the EMT induced by TNF‐α‐Smurf1‐Akt‐mTOR‐P70S6K pathway

**DOI:** 10.1111/jcmm.14420

**Published:** 2019-05-29

**Authors:** Jiajun Zhou, Hong Cheng, Zijie Wang, Hao Chen, Chuanjian Suo, Hengcheng Zhang, Jiayi Zhang, Yanhao Yang, Liang Geng, Ming Gu, Ruoyun Tan

**Affiliations:** ^1^ Department of Urology the First Affiliated Hospital, Nanjing Medical University Nanjing China

**Keywords:** Akt/mTOR/p70S6K, Bortezomib, chronic allograft dysfunction, EMT, kidney interstitial fibrosis, kidney transplantation, Smurf1

## Abstract

Allograft interstitial fibrosis was characterized by massive extracellular matrix deposition caused by activated fibroblasts and myofibroblasts. Epithelial‐mesenchymal transition (EMT) is recognized as an important source of myofibroblasts contributing to the pathogenesis of allograft interstitial fibrosis. Smad ubiquitination regulatory factor 1 (Smurf1) has been recently reported to be involved in the progression of EMT. Our study was to detect the effect of Bortezomib and Smurf1 in the EMT and allograft interstitial fibrosis. Biomarkers of EMT, as well as Smurf1, were examined in human proximal tubular epithelial cells (HK‐2) treated with tumour necrosis factor‐alpha (TNF‐α) in various doses or at various time points by Western Blotting or qRT‐PCR. We knockdown or overexpressed Smurf1 in HK‐2 cells. Furthermore, rat renal transplant model was established and intervened by Bortezomib. Allograft tissues from human and rats were also collected and prepared for HE, Masson's trichrome, immunohistochemical staining and western blotting assays. As a result, we found that TNF‐α significantly promoted the development of EMT in a time‐dependent and dose‐dependent manner through Smurf1/Akt/mTOR/P70S6K signalling pathway. More importantly, Bortezomib alleviated the progression of EMT and allograft interstitial fibrosis in vivo and in vitro by inhibiting the production of TNF‐α and expression of Smurf1. In conclusion, Smurf1 plays a critical role in the development of EMT induced by TNF‐α. Bortezomib can attenuate the Sumrf1‐mediated progression of EMT and renal allograft interstitial fibrosis, which could be suggested as a novel choice for the prevention and treatment of renal allograft interstitial fibrosis.

## 1 INTRODUCTION

Renal transplantation (RT) is the most effective therapeutic treatment for end‐stage renal disease.^1^ T‐cell mediated acute rejection was effectively reduced by immunosuppressants.^2^ However, long‐term survival rate of allografts and recipients is still limited because of the progressive deterioration of allograft function and (CAD).^3^, ^4^ Kidney interstitial fibrosis, the most important pathological lesion of CAD, was derived from the extracellular matrix deposition caused by activated fibroblasts.^5^

Epithelial‐mesenchymal transition (EMT), as a source of myofibroblasts, is characterized that epithelial cells are transformed to α‐smooth muscle actin (α‐SMA) positive myofibroblasts and lose their epithelial markers such as E‐cadherin.^6^, ^7^ EMT is regulated by various bio‐mediators such as transforming growth factor‐beta (TGF‐β), Ang II and microRNAs.^8^, ^9^, ^10^ Our previous research has shown that tumor necrosis factor‐alpha (TNF‐α) could induce the progression of EMT. HK‐2 cells lose the expression of E‐cadherin and overexpress α‐SMA and fibronectin after the treatment of TNF‐α.^11^

Smad ubiquitination regulatory factor 1 (Smurf1), an E3 ubiquitin ligase, was reported that it was involved in the EMT in lens epithelial explants, breast cancer and chronic kidney diseases (CKD) induced by TGF‐β.^12^, ^13^, ^14^ In recent studies, Smurf1 was reported that it was involved in the fibrosis of diabetic kidneys and obstructive nephropathy.^15^, ^16^ But the role of Smurf1 in the interstitial fibrosis of transplant kidney is still unknown. Depending on these reported findings, we hypothesized that Smurf1 might play a critical role in the transplant kidney interstitial fibrosis caused by EMT.

Bortezomib, a proteasome inhibitor, is used to prevent the production of alloantibody in the treatment of antibody‐mediated rejection (ABMR) through the induction of plasma cells apoptosis.^17^ It was also reported that could alleviate the fibrosis of skin, lung and CKD stimulated by TGF‐β.^18^, ^19^ However, the effect of Bortezomib in the treatment of allograft kidney interstitial fibrosis caused by EMT still remain to be determined. The aim of this study was to investigate the role and the potential molecular mechanisms of Smurf1 in the EMT induced by TNF‐α and to detect the anti‐fibrogenic effect of Bortezomib. Our results show that Smurf1 plays a critical role in regulating the EMT induced by TNF‐α. Bortezomib can attenuate the Sumrf1‐mediated progression of EMT and renal allograft interstitial fibrosis.

## 2 MATERIALS AND METHODS

### 2.1 Ethics statement

The study protocol was in accordance with the ethical standards of the Declaration of Helsinki and Istanbul. The study protocol involving human kidney tissues was approved by the local ethics committee of the First Affiliated Hospital of Nanjing Medical University. Written informed consent was obtained from all transplant recipients and nephrectomy patients.

### 2.2 Sample collection

Allograft kidney sections were obtained from 30 patients, which were collected from transplanted kidney nephrectomy or kidney biopsy of recipients. All patients, who had undergone kidney transplantation at our centre from January 2001 to October 2017 was diagnosed with CAD according to their allograft biopsy results. Patients diagnosed with CAD was classified as CAD group. In addition, 25 normal kidney samples obtained from more than 5 cm away from the tumour tissues were collected from radical nephrectomy and considered as control group. The baseline characteristics of patients in the CAD group and control group are given in Table 1.

**Table 1:** Baseline characteristics of the CAD and control groups

Clinical variables	CAD group	Control group	P value
n	30	25	
Age (years, mean ± SD)	38.13 ± 3.11	37.13 ± 2.81	NS
Male (%)	18(60%)	16(64%)	NS
BMI (kg/m2, mean ± SD)	23.29 ± 4.51	22.81 ± 4.8	NS
Transplant duration (years, range)	6.9(4.7‐10.2)		
Primary/secondary kidney transplant	30/0		
% PRA at transplant	0		
Immunosuppressive regimen			
Prednisone + MMF + Tac	15		
Prednisone + MMF + CsA	15		
Biochemical parameters			
Serum creatinine (μmol/L, mean ± SD)	520.1 ± 25.18	77.79 ± 5.13	<0.001
eGFR^a^ (min/1.73 m^2^, mean ± SD)	23.1 ± 2.99	75.12 ± 5.20	<0.001

Abbreviations: CAD: Chronic allograft dysfunction, BMI: body mass index, PRA: panel reaction antibody, MMF: mycophenolate mofetil, CsA: cyclosporine A, Tac: tacrolimus, eGFR: estimated glomerular filtration rate, SD: standard deviation, NS: no significance. ^a^eGFR was estimated by the Cockcroft‐Gault formula: eGFR = (140 ± age) 9 weight/72 9 serum creatinine 9 (0.85 if female)

### 2.3 Animals

Adult male F344 and Lewis rats (200‐250 g) were procured from Charles River Laboratories (Beijing, China). The animal centre provided the rats with the clean tap water and standard rat chow. Animal handling procedures were in accordance with guidelines published by the US National Institutes of Health, and animal ethics established by Nanjing Medical University.

### 2.4 Kidney transplantation

Orthotopic left kidney transplantation was performed between F344 and Lewis rats as previously described.^20^ Nephrectomy of the right kidney was performed 10 days after the surgery of the transplantation. The average time of cold ischaemia and warm ischaemia was 30 or 35 minutes, respectively. In case of the acute rejection, cyclosporine A (5 mg/kg bodyweight; Neoral, Novartis, Switzerland) was used once a day intraperitoneally, lasting for 14 days.

### 2.5 Pharmaceutical treatment and tissue harvest

From the first day after the kidney transplantation, Bortezomib (0.2 mg/kg; Selleck Chemicals, USA) was *iv* injected to isogeneic or allogeneic recipient rats twice a week.

At 4 weeks, 8 weeks, 12 weeks and 16 weeks, organs were harvested and divided into two parts, which was fixed in paraffin or snap‐frozen in N2 and stored at −80°C.

### 2.6 Enzyme‐linked immunosorbent assay

The levels of rat serum TNF‐α were quantified by the rat TNF‐α ELISA kit (MUTISCIENCES; China). Rat serum and cell culture medium supernatant level of TGF‐β1 were quantified by the TGF‐β1 ELISA kit (MUTISCIENCES; China). The assays were performed as described in the manufacturer's instruction.

### 2.7 Histology and morphometry

Histological analysis was performed by using haematoxylin and eosin (H&E) and Masson trichome staining. H&E and Masson trichome staining were performed as detailed elsewhere, which was used to evaluate the severity of chronic kidney rejection and the area of renal interstitial fibrosis separately.^21^ Image‐Pro Plus (Media Cybernetics, Rockville, MD) was used by two pathologists blinded to the experimental design independently to quantify the morphometric change of the kidney sections.

### 2.8 Immunohistochemistry

Immunohistochemical staining assays were performed to evaluate the expression and distribution of TNF‐α, α‐SMA, Smurf1, Fibronectin, E‐cadherin, collagen I. Paraffin sections (4 μm) were deparaffinized and rehydrated. Then, antigen retrieval was performed before primary antibody incubation, including suppression of endogenous peroxidase activity and 10% skim milk blocking. Kidney sections were incubated overnight with anti‐collagen I (1:100; Abcam, USA), anti‐Smurf1 (1:100; Santa Cruz Biotechnology, USA), anti‐ TGF‐β1 (1:100; CST, USA), anti‐ TNF‐α (1:100; CST, USA), anti‐α‐SMA (1:200; Abcam, USA), anti‐Fibronectin (1:100;Abcam, USA) and anti‐E‐cadherin (1:50; BD Biosciences, USA) primary antibodies at 4°C. After that, slices were incubated with biotinylated goat anti‐mouse/rabbit IgG (0.5 μg/mL; Abcam) for 1 hr Digital images of immunohistochemistry staining were captured and analysed by two authors independently with the help of a light microscope (ECLIPSE 80i; Nikon).

### 2.9 Indirect immunofluorescence double‐staining assay

Immunofluorescence double‐staining assay were performed as previously described, using antibodies against TGF‐β1 (1:100; CST, USA), α‐SMA (1:200; Abcam, USA), E‐cadherin (1:50; BD Biosciences, USA).^21^ The fluorescence intensities of TGF‐β1, E‐cadherin and α‐SMA were performed using Image‐Pro Plus (Media Cybernetics, USA).

### 2.10 Renal function assessment

Concentrations of rat blood creatinine and urea nitrogen were tested by instructions of manufacturer of the kits (Jiancheng, Beijing, China).

### 2.11 Quantitative real‐time PCR analysis

Total RNA was extracted from cells with the RNA extraction kits (TIANGEN, Beijing, China). cDNA was synthesized with a PrimeScriptTMRT reagent kit (TaKaRa Biotechnology, Japan). qRT‐PCR was performed with a SYBR Green PCR kit (TaKaRa Biotechnology) on a DNA Engine Opticon 2 System (BioRad laboratories, Hercules, CA). The specific primers used were as follows:

Smurf1: 5′‐ CTACCAGCGTTTGGATCTAT‐3′ (F)

5′‐ TGTCTCGGGTCTGTAAACT‐3′ (R);

CDH1: 5′‐CGAGAGCTACACGTTCACGG‐3′ (F)

5′‐GGGTGTCGAGGGAAAAATAGG‐3′(R);

ACTA2: 5′‐AAAAGACAGCTACGTGGGTGA‐3′ (F)

5′‐GCCATGTTCTATCGGGTACTTC‐3′(R);

Fibronectin1: 5′‐ CGGTGGCTGTCAGTCAAAG‐3′(F)

5′‐AAACCTCGGCTTCCTCCATAA‐3′(R);

Actin: 5′‐TGACGTGGACATCCGCAAAG‐3′ (F)

5′‐CTGGAAGGTGGACAGCGAGG‐3′ (R).

mRNA expression was normalized to b‐actin expression. Every experiment described previously was repeated at least three times.

### 2.12 Western blot analysis

Western blotting was performed according to the protocol described by Liu et al^22^ Total proteins were extracted from cells, human tissues or rat tissues. Then, western blotting was conducted by incubating the protein with primary antibodies. The primary antibodies were as followed: anti‐GAPDH (1:1000; CST, USA), anti‐E‐cadherin (1:1000; BD Biosciences, USA), anti‐α‐SMA (1:1000; Abcam, USA), anti‐fibronectin (1:1000; BD Biosciences, USA), anti‐Smurf1 (1:1000; Santa Cruz Biotechnology, USA), anti‐Akt (1:1000; CST, USA), anti‐phospho‐Akt (1:1000; CST, USA), anti‐mTOR (1:1000; CST, USA), anti‐P70S6K (1:1000; CST, USA), anti‐phospho‐mTOR (1:1000; CST, USA), anti‐phospho‐P70S6K(1:1000; CST, USA).Then, the strips were incubated by an anti‐rabbit or anti‐mouse secondary antibody(1:2000; CST, USA). GAPDH was used to measure the relative abundance of proteins as an internal reference. The intensity of the protein signals was detected by the NIH image analysis software.

### 2.13 Cell culture, treatment and transfection

HK‐2s were cultured in Dulbecco's modified Eagle's medium (DMEM)/F12 medium containing 10% foetal bovine serum and 1% penicillin‐streptomycin in a humidified atmosphere containing 5% CO2 at 37°C. For TNF‐α treatment, the cells were starved without serum overnight and then treated with 50 ng/mL TNF‐α for 0, 0.5, 1, 2, 4, 6, 8, 12, 24, 48 hours or 10, 20, 50, 100 ng/mL TNF‐α for 24 hours. MK‐2206 (10 μM) was previously given to HK‐2 cells for 0.5 hour before the treatment of TNF‐α, in order to determine the effects of Akt pathway on the Smurf1‐meditated EMT. To evaluate the anti‐fibrogenic effect, cells were divided into five groups and then starved without serum overnight. Four of them were co‐treated with TNF‐α (50 ng/mL) and Bortezomib (0, 10, 30, 50 ng/mL) for 24 hours.

Construction and verification of stable Smurf1‐knockdown and Smurf1‐overexpression cell lines:HK‐2 cells were inoculated into 12‐well plates, then added with 1.5 μL Smurf1 short hairpin ribonucleic acid (shRNA) viruses or 2.0 μL Smurf1 overexpressing viruses. Control viruses were added at the same time. The cells were further cultured with medium containing 2 μL polybrene for 6 hours. After that, medium was removed and cells were cultured with non‐polybrene medium until 48 hours. The infection rate of viruses was observed under a fluorescence microscope (Olympus, Japan), and puromycin was added to kill the uninfected cells. The expression level of Smurf1 protein was determined by western blotting.

### 2.14 Statistical analysis

All data were presented as the mean ± SD values determined from three independent experiments. After demonstration of homogeneity of variance with Bartlett test, inter‐group comparisons were made using one‐way analysis of variance (ANOVA). Multiple means were compared using Tukey's test. The differences between two groups were determined by Student *t* test. Values of *P* < 0.05 were considered statistically significant. All the assays were repeated at least three times independently.

## 3 RESULTS

### 3.1 Smurf1 exhibits remarkably high expression in the kidney tissues of renal interstitial fibrosis from CAD patients

**Figure 1:**
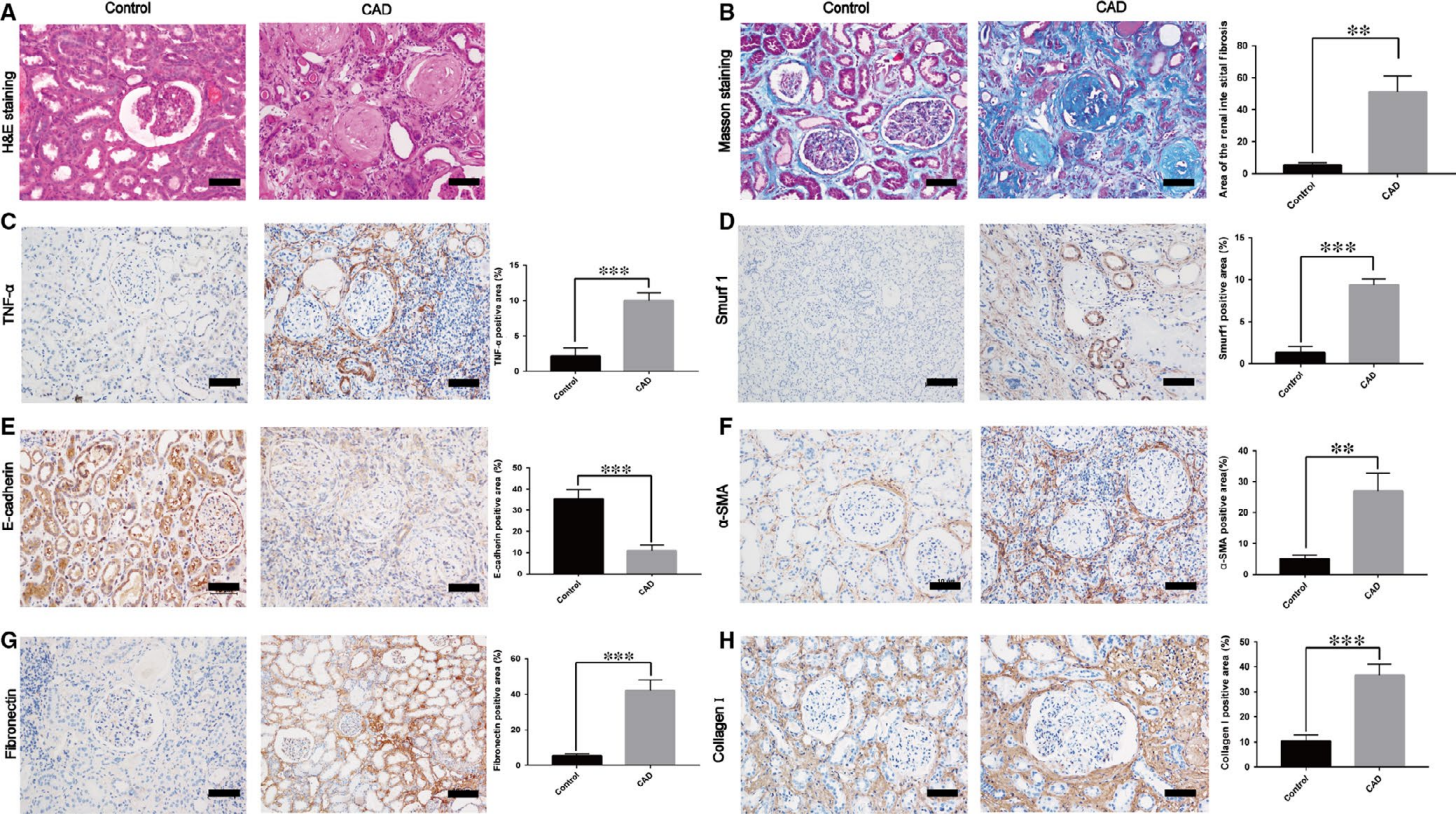
Smurf1 exhibits remarkably high expression in CAD patients’ kidney. (A‐B) Representative tissue sections from control group (n = 25) and CAD group (n = 30) were stained with HE (A) and Masson staining (B). Bar = 10 μm. (B) Quantitative analysis of the fibrosis intensity of kidney sections stained with Masson‐Trichrome was performed. (C‐H) Representative immunohistochemistry staining and quantitative analysis of TNF‐α, Smurf1, E‐cadherin, α‐SMA, fibronectin, collagen I. Bar = 10μm. Data are presented as the mean ± SD value of three independent experiments. **P < 0.01 vs control group. ***P < 0.001 vs control group

In this study, we used human specimens to confirm the high expression of Smurf1 in the CAD patients with allograft renal interstitial fibrosis. Histological studies with HE and Masson staining revealed significant renal interstitial fibrosis from CAD group (n = 30) to control group (n = 25) (Figure 1A,B). Moreover, the results of immunohistochemistry staining showed that the expression of TNF‐α, α‐SMA, collagen I, fibronectin and Smurf1 was significantly higher and the expression of E‐cadherin was remarkably lower in the CAD group (Figure 1C‐H).

### 3.2 Progression of EMT in HK‐2 cells could be stimulated by TNF‐α

**Figure 2:**
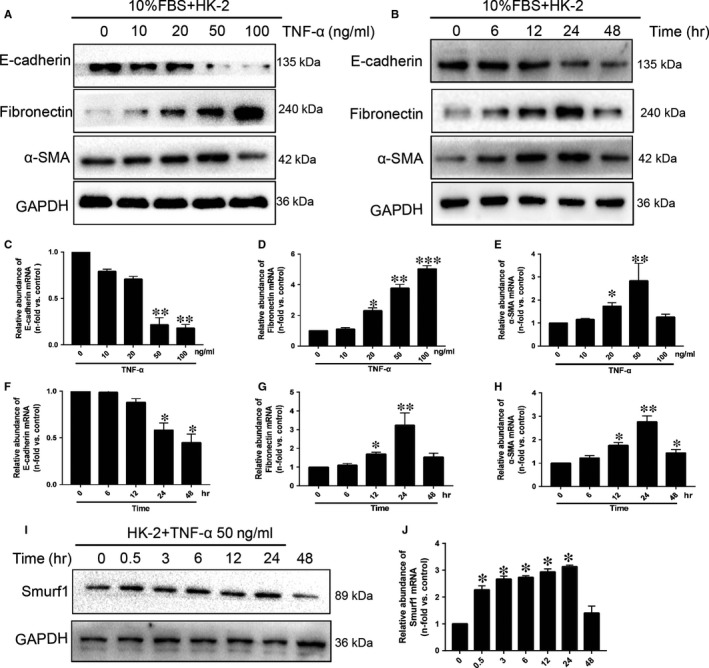
Smurf1 is remarkably increased in the EMT stimulated by TNF‐α. TNF‐α promotes Smurf1, α‐SMA and fibronectin expression and suppresses E‐cadherin expression in the HK‐2s. Equal amounts of protein from whole cell lysates were analysed by western blotting with antibodies against α‐SMA, fibronectin, E‐cadherin and GAPDH after stimulation of HK‐2s with various concentrations of TNF‐α for 24 hours (A) or incubation of HK‐2s with 50ng/mL TNF‐α for the indicated time points (B). (C‐J) HK‐2s were stimulated with various concentrations of TNF‐α for 24 hours (C‐E) or with 50ng/mL TNF‐α for the indicated time points(F‐J). (I) Representative western blot of Smurf1 indicated protein expression of Smurf1 has remarkably increased when HK‐2s was stimulated for 0.5 h. Total RNA was isolated and subjected to quantitative real‐time PCR to detect the gene expression of α‐SMA, fibronectin, E‐cadherin and Smurf1. Relative abundance of mRNAs is presented as the mean ± SD value of three independent experiments. The PCR results were in agreement with the western blot results. *P < 0.05, **P < 0.01, ***P < 0.001 vs the control group

To investigate the pathogenesis of EMT stimulated by TNF‐α, we stimulated the HK‐2 cells with different concentrations of TNF‐α(0,10,20,50 ng/mL) for 24 hours. TNF‐α up‐regulated the protein and mRNA expression of fibronectin and α‐SMA, leading to a dose‐dependent decline in the expression of E‐cadherin at the same time (Figure 2A,C‐E). This effect was peaked while the HK‐2 cells were treated by 50 ng/mL TNF‐α, and was reversed at the concentration of 100 ng/mL TNF‐α. Time‐dependent studies were performed to confirm that the protein and mRNA expression of fibronectin and α‐SMA. This effect was at the peak when HK‐2 cells were treated by 50 ng/mL TNF‐α for 24 hours (Figure 2B,G,H). TNF‐α down‐regulated the expression of E‐cadherin in a time‐dependent manner, and it was confirmed by the results of western blot and qRT‐PCR (Figure 2B,F). Double‐staining immunofluorescence of α‐SMA and E‐cadherin confirmed our outcome (Figure S1A‐C). Protein and mRNA expression of Smurf1 at different time points was determined by western blot and qRT‐PCR. Compared with 0h group, the relative protein and mRNA abundance of Smurf1 was remarkably increased after TNF‐α treatment for 0.5 hours (Figure 2I,J). This effect was at the peak when HK‐2 cells were treated by TNF‐α for 24 hours. All these changes indicated that HK‐2 cells underwent transition into mesenchymal cells after the treatment of TNF‐α, and Smurf1 was up‐regulated in this progression.

### 3.3 Smurf1 plays a critical role in the EMT induced by TNF‐α

**Figure 3:**
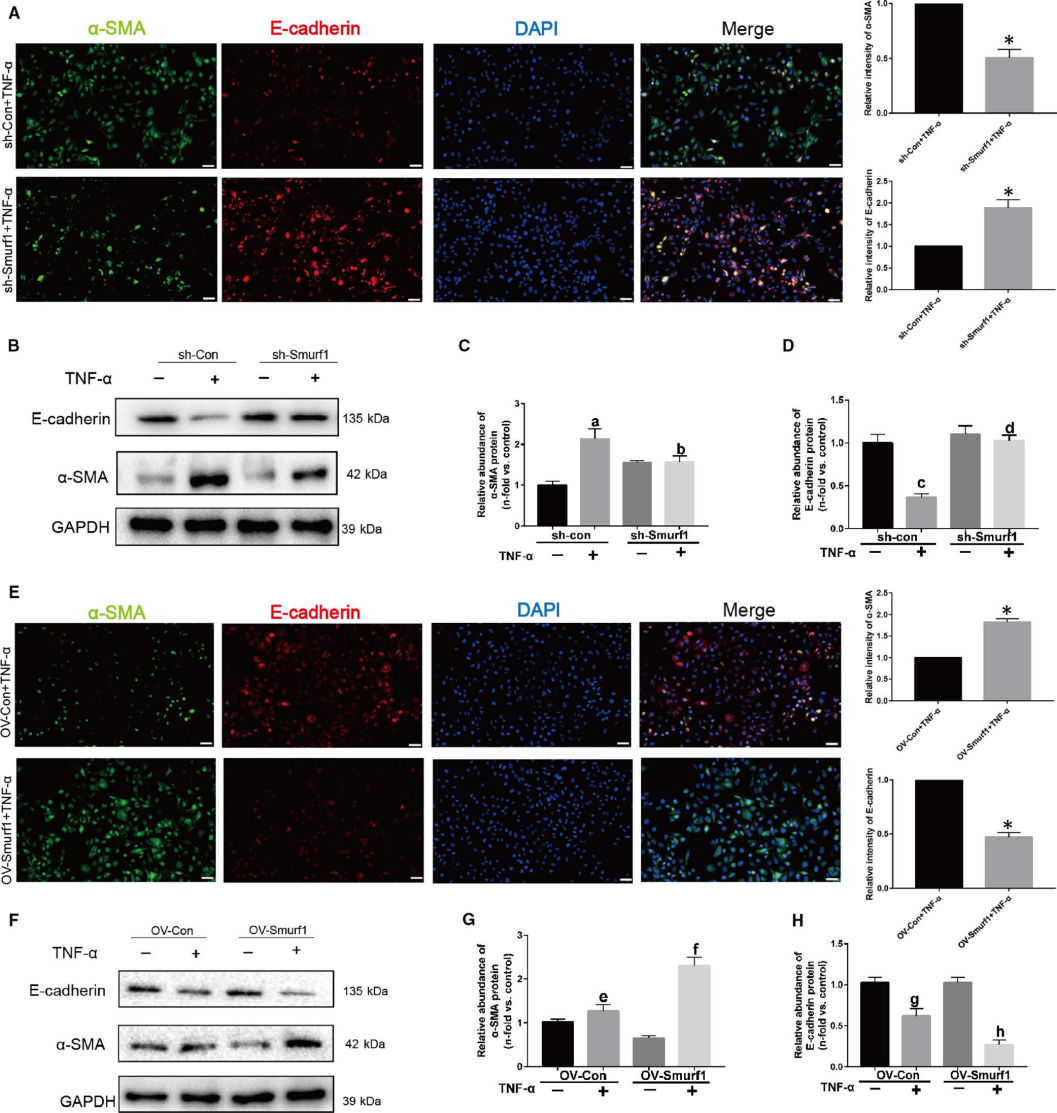
Smurf1 plays a critical role in the EMT induced by TNF‐α. (A‐D) Knockdown of Smurf1 significantly attenuated the progression of EMT induced by TNF‐α. (A) Representative immunofluorescence staining of α‐SMA and E‐cadherin indicates that knockdown of Smurf1 significantly reduces the expression of α‐SMA and inhibits the loss of E‐cadherin. Quantitative analysis of relative intensity of E‐cadherin and α‐SMA was performed. Bar = 50μm (B) Representative western blot of E‐cadherin and α‐SMA. (C‐D) Quantitative analysis of relative protein abundance of E‐cadherin and α‐SMA. (E‐H) Overexpression of Smurf1 significantly promotes the progression of EMT induced by TNF‐α. (E) Representative immunofluorescence staining of α‐SMA and E‐cadherin indicates overexpression of Smurf1 significantly increased the expression of α‐SMA and promoted the loss of E‐cadherin. Quantitative analysis of relative intensity of E‐cadherin and α‐SMA was performed. Bar = 50μm. (F) Representative western blot of E‐cadherin and α‐SMA. (G‐H) Quantitative analysis of relative protein abundance of E‐cadherin and α‐SMA. The relative abundance of proteins and relative intensity of E‐cadherin and α‐SMA was presented as the mean ± SD values of three independent experiments. ^a^ *P* < 0.05 vs sh‐con cells without TNF‐α treatment, ^b^ *P* < 0.05 vs sh‐con cells stimulated by TNF‐α, ^c^ *P* < 0.01 vs sh‐con cells without TNF‐α treatment, ^d^ *P* < 0.01 vs sh‐con cells stimulated by TNF‐α, ^e^ *P* < 0.05 vs sh‐con cells without TNF‐α treatment, ^f^ *P* < 0.05 vs sh‐con cells stimulated by TNF‐α, ^g^ *P* < 0.05 vs sh‐con cells without TNF‐α treatment, ^h^ *P* < 0.05 vs sh‐con cells stimulated by TNF‐α, *P < 0.05 vs sh‐con cells or ov‐con cells stimulated by TNF‐α

To confirm the role of Smurf1 in the EMT induced by TNF‐α, we examined the effect of Smurf1 silencing on these events by constructing Smurf1 stable knockdown HK‐2 cells. As shown in the Figure 3A, relative intensity of α‐SMA in sh‐Smurf1 group stimulated by TNF‐α was significantly less than sh‐con group according to the outcome of immunofluorescence. However, the relative intensity of E‐cadherin was remarkably higher than sh‐con group. Knockdown of Smurf1 blocked the expression of α‐SMA, up‐regulated the expression of E‐cadherin and alleviated the progression of EMT (Figure 3B‐D). When the Smurf1 was overexpressed, the progression of EMT was significantly promoted (Figure 3E‐H). These data supported our conclusion that Smurf1 plays a critical role in the EMT of HK‐2 cells.

### 3.4 Bortezomib alleviates the Smurf1‐meditated EMT of HK‐2 cells by inhibiting TNF‐α‐Akt‐mTOR‐P70S6K pathway

**Figure 4:**
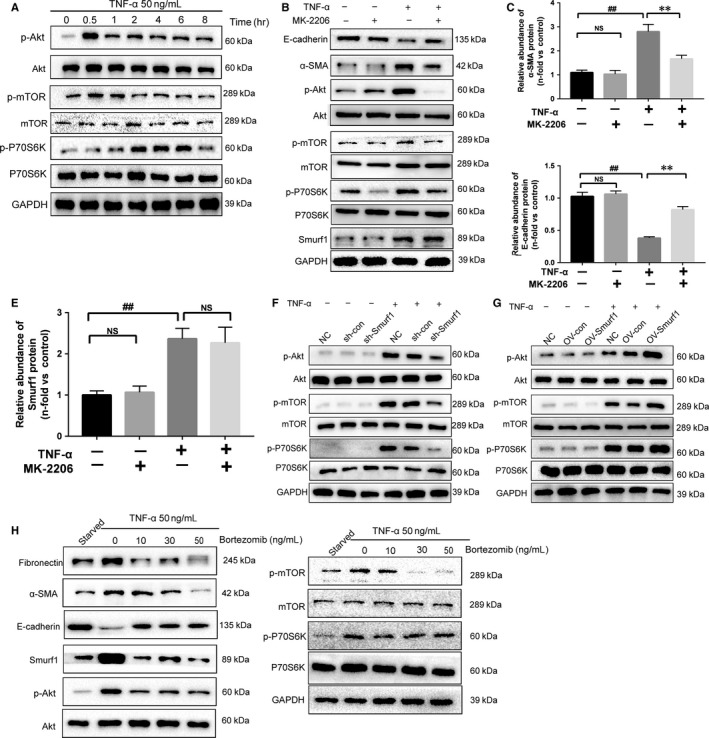
Bortezomib alleviates the Smurf1‐meditated EMT of HK‐2 cells by inhibiting TNF‐α‐Akt‐mTOR‐P70S6K pathway. TNF‐α up‐regulates α‐SMA expression in HK‐2s through the TNF‐α‐Smurf1‐Akt‐mTOR‐P70S6K signalling pathways. The anti‐EMT role of Bortezomib was through inhibiting the expression of Smurf1. (A) The Akt‐mTOR‐P70S6K pathway is activated after the treatment of TNF‐α. (B) The Akt inhibitor MK‐2206 reduced the α‐SMA expression and restored the expression of E‐cadherin. But MK‐2206 showed little effects on the expression of Smurf1. (C‐E) Quantitative analysis of relative protein abundance of α‐SMA, E‐cadherin and α‐SMA was performed. The relative abundance of proteins was presented as the mean ± SD values of three independent experiments. ^##^ *P* < 0.01 vs normal HK‐2 cells. **P < 0.01 vs normal HK‐2 cells treated with TNF‐α and MK‐2206. (F) OV‐Smurf1 cells showed a significant increase of phosphorylated Akt, mTOR and P70S6K than OV‐con cells after the treatment of TNF‐α. (G) sh‐Smurf1 cells showed a remarkable decrease of phosphorylated Akt, mTOR and P70S6K than sh‐con cells after the treatment of TNF‐α. Bortezomib alleviated the progression of EMT by blocking the TNF‐α‐Smurf1‐Akt‐mTOR‐P70S6K pathway. The effect of Bortezomib was to remarkably decrease the expression of Smurf1

To evaluate the signalling pathways involved in TNF‐α‐induced EMT in HK‐2 cells, we examined phosphorylation of factors involved in the canonical signalling pathways by western blotting. We observed the activation of the Akt‐mTOR‐P70S6K signalling pathways when HK‐2 cells were treated with TNF‐α (Figure 4A). The Akt inhibitor MK‐2206 could reduce the expression of α‐SMA and restore the expression of E‐cadherin (Figure 4B‐C). Immunofluorescence staining of α‐SMA and E‐cadherin confirmed our conclusions (Figure S2A‐C). On the other hand, MK‐2206 could not reduce the high expression of Smurf1 stimulated by TNF‐α (Figure 4B,D). The Smurf1 stable knockdown HK‐2 cells treated by TNF‐α showed remarkably less phosphorylation of Akt, mTOR and P70S6K than sh‐con cells (Figure 4F). This effect was reversed between the Smurf1 stable overexpression HK2 cells and OV‐con cells (Figure 4G). These results revealed that the EMT induced by TNF‐α in HK‐2 cells was through the Smurf1‐Akt‐mTOR‐P70S6K pathway. To evaluate the effect of Bortezomib, HK‐2 cells were co‐treated with 50 ng/mL TNF‐α and different concentrations of Bortezomib for 24 hours. The effect that Bortezomib alleviated the EMT of HK‐2 cells was at the peak when HK‐2 cells were co‐treated by Bortezomib(50 ng/mL) and TNF‐α(50 ng/mL) for 24 hours. Bortezomib attenuated the EMT and fibrosis by reducing the expression of Smurf1 (Figure 4G). The protein expression of Smurf1, α‐SMA and fibronectin was remarkably reduced by Bortezomib, while the expression of E‐cadherin was restored to a limited extent (Figure 4G). The immunofluorescence staining of α‐SMA and E‐cadherin also determined Bortezomib could attenuated the EMT of HK‐2 cells induced by TNF‐α (Figure S2A‐C).

### 3.5 Bortezomib ameliorates the inflammatory response and renal interstitial fibrosis in rat allograft kidney after transplantation

**Figure 5:**
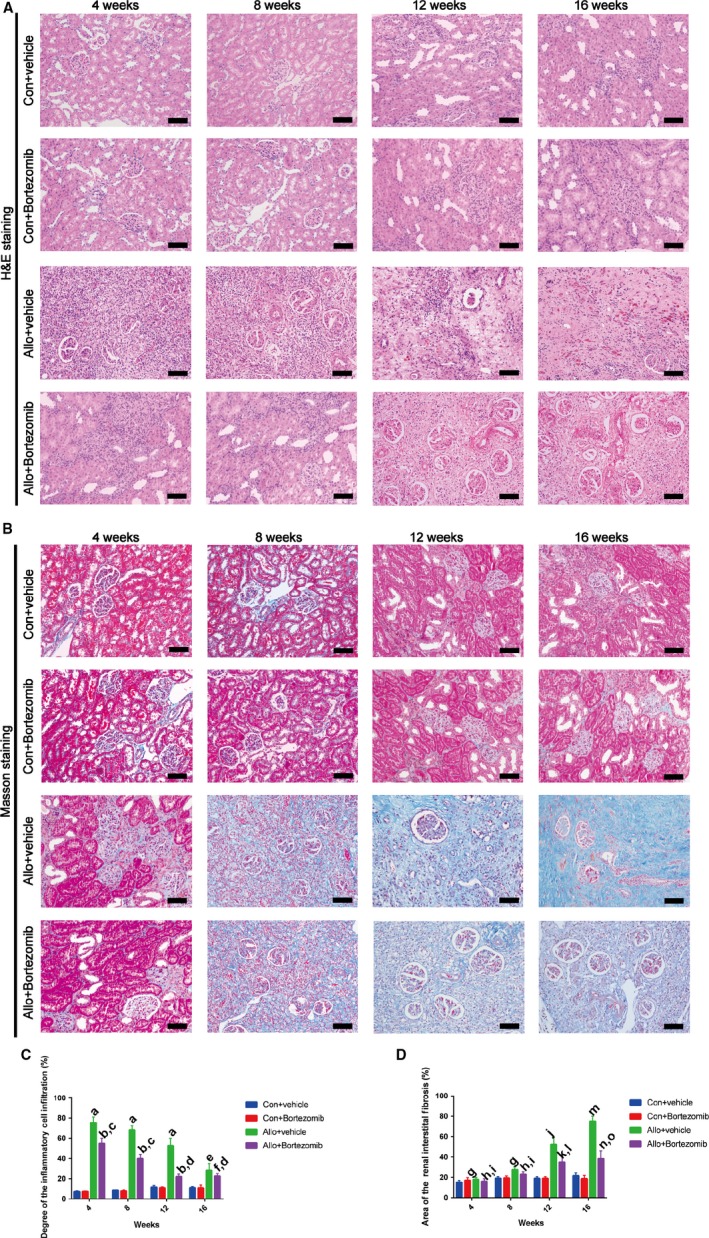
Bortezomib ameliorates the inflammatory response and renal interstitial fibrosis in rat allograft kidney after transplantation. After 8 weeks of kidney transplantation, the allograft kidney developed into renal interstitial fibrosis. The renal interstitial fibrosis was first occurred at 8 weeks after transplantation. After 16 weeks of kidney transplantation, the area of fibrosis was up to 75%. The area of inflammatory cell infiltration was up to almost 80% after 4 weeks of transplantation. Bortezomib ameliorates the inflammatory response and renal interstitial fibrosis in rat allograft kidney after transplantation. (A‐B) Representative HE (A) and Masson staining (B) of syngeneic and allogeneic kidney at different periods. Bar = 10μm. (C‐D) Quantitative analysis of the inflammatory cell infiltration (C) and the fibrosis intensity of kidney sections (D) was performed. Data of lesion area are expressed as the mean ± SD of each group (n = 3) from 3 separate experiments. ^a^ *P* < 0.01 vs syngeneic recipients treated with vehicle. ^b^ *P* < 0.01 vs allogeneic recipients treated with vehicle. ^C^ *P* < 0.01 vs syngeneic recipients treated with Bortezomib. ^d^ *P* < 0.05 vs syngeneic recipients treated with Bortezomib. ^e^ *P* < 0.05 vs syngeneic recipients treated with vehicle. ^f^ *P* < 0.05 vs allogeneic recipients treated with vehicle. ^g^ *P* < 0.05 vs syngeneic recipients treated with vehicle. ^h^ *P* > 0.05 vs allogeneic recipients treated with vehicle. ^i^ *P* < 0.05 vs syngeneic recipients treated with Bortezomib. ^j^ *P* < 0.05 vs syngeneic recipients treated with vehicle. ^k^ *P* < 0.05 vs allogeneic recipients treated with vehicle. ^l^ *P* < 0.05 vs syngeneic recipients treated with Bortezomib. ^m^ *P* < 0.01 vs syngeneic recipients treated with vehicle. ^n^ *P* < 0.01 vs allogeneic recipients treated with vehicle. ^o^ *P* < 0.01 vs syngeneic recipients treated with Bortezomib.

In this study, we applied Bortezomib to explore the role of the proteasome inhibitor in ameliorating renal interstitial fibrosis in allograft kidney after transplantation. Rat renal transplant model was established by transplantation from F344 rat donors to Lewis rat recipients. 0.2 mg/kg Bortezomib was injected to the allo‐recipient rats through tail vein every three days. After 4 weeks of transplantation, allograft kidney was infiltrated with inflammatory cells and the lesion area was up to almost 80% according to the results of HE staining (Figure 5A, 5C). Bortezomib remarkably attenuated the degrees of inflammatory cells infiltration at 4,8,12,16 weeks (Figure 5A). Renal interstitial fibrosis of allograft kidney, firstly occurred at 8 weeks after kidney transplantation. The lesion area of interstitial fibrosis was dramatically reduced by the treatment of Bortezomib after 8 weeks of kidney transplantation (Figure 5B, 5D). Histological studies revealed that Bortezomib could ameliorated the inflammatory response and renal interstitial fibrosis in allograft kidney after transplantation.

### 3.6 Bortezomib alleviated the Smurf1‐meditated EMT and renal interstitial fibrosis by reducing the secretion of TNF‐α in kidney transplantation

**Figure 6:**
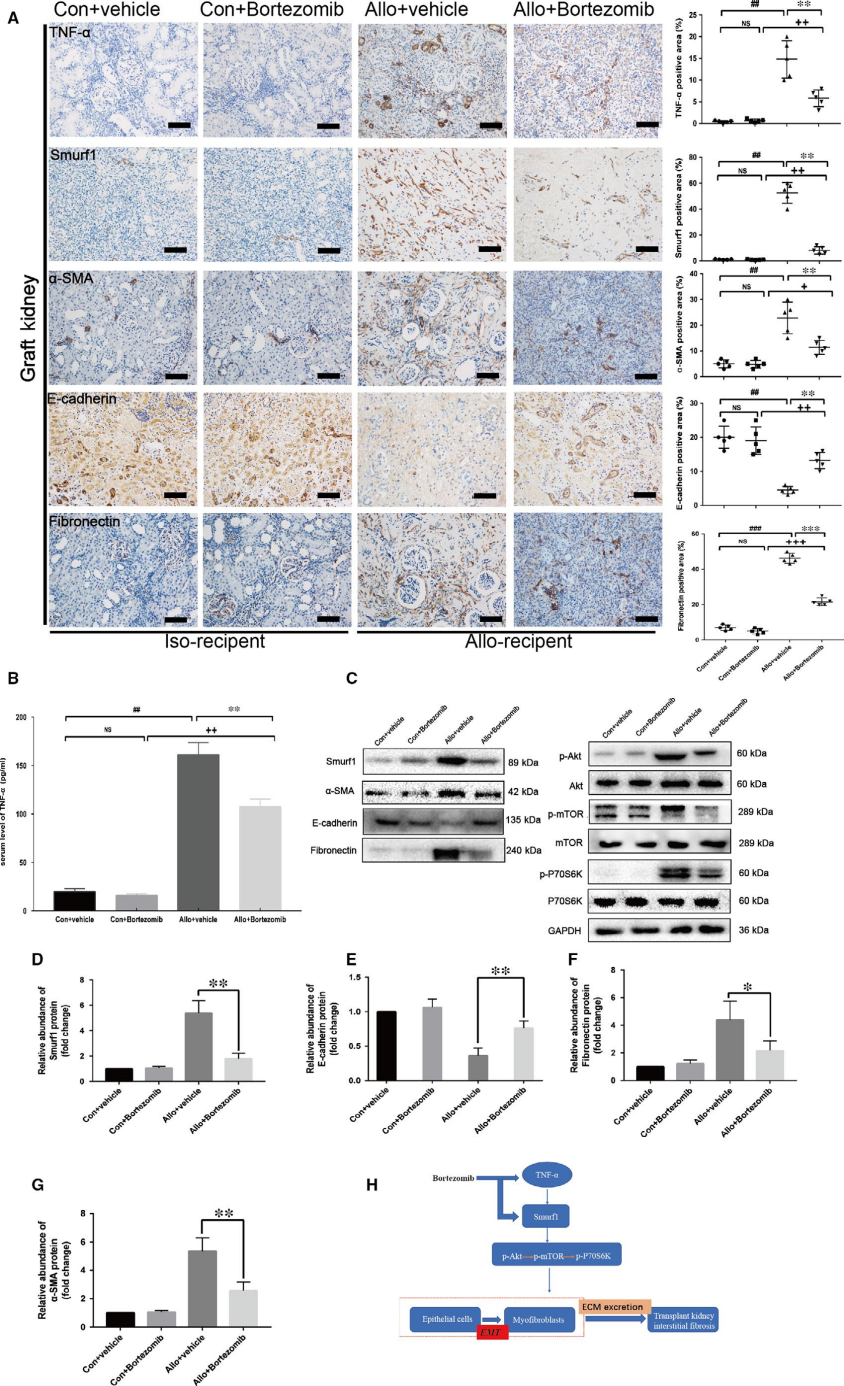
Bortezomib alleviated the Smurf1‐meditated EMT and renal interstitial fibrosis by reducing the secretion of TNF‐α in kidney transplantation. Our *vivo* findings revealed that TNF‐α‐Smurf1‐Akt‐mTOR‐P70S6K pathway was critical in the progression of EMT. Rat model of allograft kidney interstitial fibrosis was constructed to confirm our *vivo* findings. After treatment of Bortezomib for 12 weeks, kidney tissue sections were stained with immunohistochemistry. Protein, extracted from rat kidney, was subjected to western blot. Serum level of TNF‐α was evaluated by ELISA kit. (A) Representative immunohistochemistry staining and quantitative analysis of TNF‐α, Smurf1, E‐cadherin, α‐SMA, fibronectin was performed. Bar = 10μm. Data are representative images or are expressed as individual spots per animal with means of each group (n = 5) from 3 separate experiments. (B) Outcomes of ELISA assays revealed that Bortezomib reduced the secretion of TNF‐α. (C) Representative western blot of Smurf1, E‐cadherin, α‐SMA, fibronectin and Akt‐mTOR‐P70S6K pathway confirmed our vitro findings. (D‐G) Quantitative analysis of relative protein abundance of Smurf1, E‐cadherin, α‐SMA, fibronectin was performed. Data are expressed as the mean ± SD of each group (n = 3) from 3 separate experiments (H) A model is proposed to illustrate the fibrotic mechanism involved in EMT induced by TNF‐α in the pathogenesis of allogeneic kidney interstitial fibrosis and the therapeutic mechanism of Bortezomib. ^##^ *P* < 0.01 vs syngeneic recipients treated with vehicle. ^###^ *P* < 0.001 vs syngeneic recipients treated with vehicle. *P < 0.05 vs allogeneic recipients treated with Bortezomib. **P < 0.01 vs allogeneic recipients treated with Bortezomib. ***P < 0.001 vs allogeneic recipients treated with Bortezomib. ^+^ *P* < 0.05 vs syngeneic recipients treated with Bortezomib. ^++^ *P* < 0.01 vs syngeneic recipients treated with Bortezomib. ^+++^ *P* < 0.001 vs syngeneic recipients treated with Bortezomib

The outcome of immunohistochemistry assay revealed the remarkably high expression of TNF‐α and Smurf1 in allo‐recipient group (Figure 6A). After the treatment of 0.2 mg/kg Bortezomib for 12 weeks, the positive area of TNF‐α and Smurf1 in allograft kidney was dramatically decreased (Figure 6A). The results of immunohistochemistry assay also indicated that Bortezomib could down‐regulate the expression of α‐SMA, fibronectin and restore the expression of E‐cadherin in the rat allograft kidney (Figure 6A). Bortezomib could significantly decreased the serum TNF‐α level determined by the ELISA assay (Figure 6B). Western blot confirmed the outcome of immunohistochemistry assay (Figure 6C‐G). The anti‐fibrosis effect of Bortezomib was not only to block the TNF‐α‐Smurf1‐Akt‐mTOR‐P70S6K pathway by reducing the expression of Smurf1, but also to decrease the secretion of inflammatory cytokines TNF‐α (Figure 6H).

### 3.7 Bortezomib prevents allograft renal function impairment and prolongs survival of allogeneic recipients

**Figure 7:**
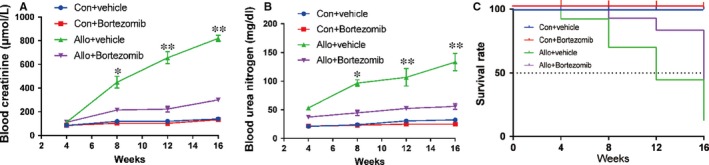
Bortezomib prevents allograft renal function impairment and prolongs survival of allogeneic recipients. The native right kidneys of recipient rats were resected 14 days after left kidney transplantation. (A‐B) The blood creatinine and urea nitrogen were continuously increased in allogeneic recipients treated with vehicle. Treatment of Bortezomib remarkably prohibited the increases of blood creatinine and urea nitrogen. (C) The survival rate curve showed that Bortezomib significantly increased the long‐term survival of allogeneic recipients. Data are expressed as the mean ± SD of each group from three separate experiments. *P < 0.05 vs allogeneic recipients treated with vehicle. **P < 0.01 vs allogeneic recipients treated with vehicle. Log‐rank test showed the Bortezomib significantly increased the survival rate of allogenic recipients. *P* < 0.05 vs allogeneic recipients treated with vehicle

In order to test the impact of Bortezomib on allograft renal function and long‐term survival of allogeneic recipients, the native right kidneys of recipients were removed 2 weeks after left kidney transplantation. One week after the resection of right kidney, the renal function including the blood concentration of creatinine and urea nitrogen, started to be continuously deteriorated until their death. However, the increase of blood creatinine and urea nitrogen was inhibited and they remained at a relatively low level by the treatment of Bortezomib (Figure 7A‐B). The survival rate of allogeneic recipients was enhanced by the treatment of Bortezomib (Figure 7C). Allogeneic recipients treated with Bortezomib enjoyed a higher survival rate compared with allo‐recipients treated by vehicle at both 12 and 16 weeks.

## 4 DISCUSSION

In present study, we reported that Bortezomib could attenuate the fibrosis of renal interstitial in kidney transplantation via regulating the EMT induced by TNF‐α‐Smurf1‐Akt‐mTOR‐P70S6K pathway. The effects of Bortezomib are not only to block the progression of the EMT induced by TNF‐α‐Smurf1‐Akt‐mTOR‐P70S6K, but also to reduce the secretion of TNF‐α. To our best knowledge, it is the first study that elucidate the role and underlying mechanism of Bortezomib, which could attenuate the fibrosis of renal interstitial in kidney transplantation. Smurf1, as a key of the EMT and renal interstitial fibrosis induced by TNF‐α, plays a critical role in this progression. Our results offer novel insights into the treatment and prevention of allograft renal interstitial fibrosis. Bortezomib could be a new way in the treatment and prevention of transplant renal interstitial fibrosis. Furthermore, Smurf1 could provide us with a new drug target of the treatment and prevention of transplant kidney interstitial fibrosis.

TGF‐β1 has been widely used in the induction of EMT in HK‐2 cells. Our previous research confirmed TNF‐α also could promote the progression of EMT in HK‐2 cells. Immunohistochemistry staining of TGF‐β1 revealed the expression of TGF‐β1 in CAD group was remarkably higher than Control group (Figure S1A‐B). Bortezomib was reported that it could attenuate renal fibrosis in mice via the suppression of TGF‐β1 in UUO model.^23^ Our vivo study in rats showed that Bortezomib could not effectively reduce the expression and secretion of TGF‐β1in allograft kidney (Figure S3C‐E). However, the increase expression of TNF‐α in allograft and secretion of serum TNF‐α could be prevented by Bortezomib treatment. We hypothesized that Bortezomib exerted its anti‐fibrogenic effects though resistance of biological effects of TNF‐α in transplant kidneys.

In order to determine the relationship between TNF‐α, Smurf1 and EMT in the transplant kidneys, our *vitro* findings revealed that the progression of fibrosis and EMT of HK‐2 was induced by the TNF‐α‐Smurf1‐Akt‐mTOR‐P70S6K pathway. In our *vitro* study, overexpression of Smurf1 without the stimulation of TNF‐α could not lead to the progression of EMT in HK‐2 cells. Unlike TNF‐α and TGF‐β1，Smurf1 served as a regulatory factor rather than inducer of the EMT progression in HK‐2 cells.

Furthermore, we found the anti‐fibrogenic effects of Bortezomib, which alleviated the fibrosis and EMT of HK‐2 cells by downregulating the expression of Smurf1. Bortezomib was reported that it prevents oncogenesis and bone metastasis of prostate cancer by inhibiting Smurf1 expression.^24^ However, its mechanism of action is not fully understood. miR‐424(322) induced the downregulation of Smurf1 in pulmonary arterial hypertension.^25^ Bortezomib could modulate various miRNA level such as microRNA‐17‐5p.^26^ The potential mechanisms of Bortezomib inhibiting the expression of Smurf1 maybe that Bortezomib promotes the expression of inhibitory miRNA, such as miR‐424(322). Smurf1, as a E3 Ubiquitin Ligases, could degrade the key protein in the TGF‐β1 induced pathway.^27^, ^28^ PTEN, an upstream molecule of PI3K‐Akt‐mTOR pathway, could dephosphorylate the PI3K to down‐regulate the activation of PI3K‐Akt‐mTOR pathway.^29^, ^30^ WWP2, another E3 Ubiquitin Ligases, could degrade PTEN by ubiquitination pathway to suppress tumourigenesis.^31^ The mechanism that Smurf1 down‐regulated the Akt‐mTOR‐P70S6K pathway could be the degradation of PTEN.

To confirm the anti‐fibrogenic effect of Bortezomib, our *vivo* findings revealed that Bortezomib could reduce the inflammatory response and renal interstitial fibrosis in allograft kidney. Bortezomib blocked the progression of EMT induced by TNF‐α‐Smurf1‐Akt‐mTOR‐P70S6K. Another positive effect of Bortezomib was to reduce the secretion of TNF‐α. The treatment of Bortezomib could prolong the survival of allo‐recipients. Bortezomib, as a proteasome inhibitor, was used to prevent the production of donor specific antibody in treatment of ABMR. The anti‐fibrogenic effect and mechanism of Bortezomib was never reported in transplant kidney. So Bortezomib could be a potential therapeutic drug in prevention and treatment of transplant kidney fibrosis.

Taken together, the current studies proved that Bortezomib attenuates the fibrosis of renal interstitial and Smurf1‐meditated EMT induced by TNF‐α‐Akt‐mTOR‐P70S6K pathway. The results of our study provide novel insight into Smurf1 and Bortezomib. Smurf1 could be an additional target for the treatment and prevention of kidney interstitial fibrosis and CAD in kidney transplant recipients. Bortezomib can attenuate the Sumrf1‐mediated progression of EMT and renal allograft interstitial fibrosis, which could be suggested as a novel choice for the prevention and treatment of renal allograft interstitial fibrosis.

## DISCLOSURE STATEMENT

The authors confirm that there are no conflicts of interest.

## AUTHORS CONTRIBUTIONS

Study design: M Gu and RY Tan; Data Collection: JJ Zhou, H Cheng, ZJ Wang and H Chen; Software material: H Cheng, CJ Suo; Data Analysis: JY Zhang, HC Zhang，YH Yang and L Geng; Manuscript preparation: JJ Zhou, H Cheng and ZJ Wang.
