# Supplementary figures and images for: Bortezomib attenuates renal interstitial fibrosis in kidney transplantation via regulating the EMT induced by TNF‐α‐Smurf1‐Akt‐mTOR‐P70S6K pathway
Source: J Cell Mol Med. 2019 May 29;23(8):5390–402. doi: 10.1111/jcmm.14420 (PMC6653435; doi:10.1111/jcmm.14420)

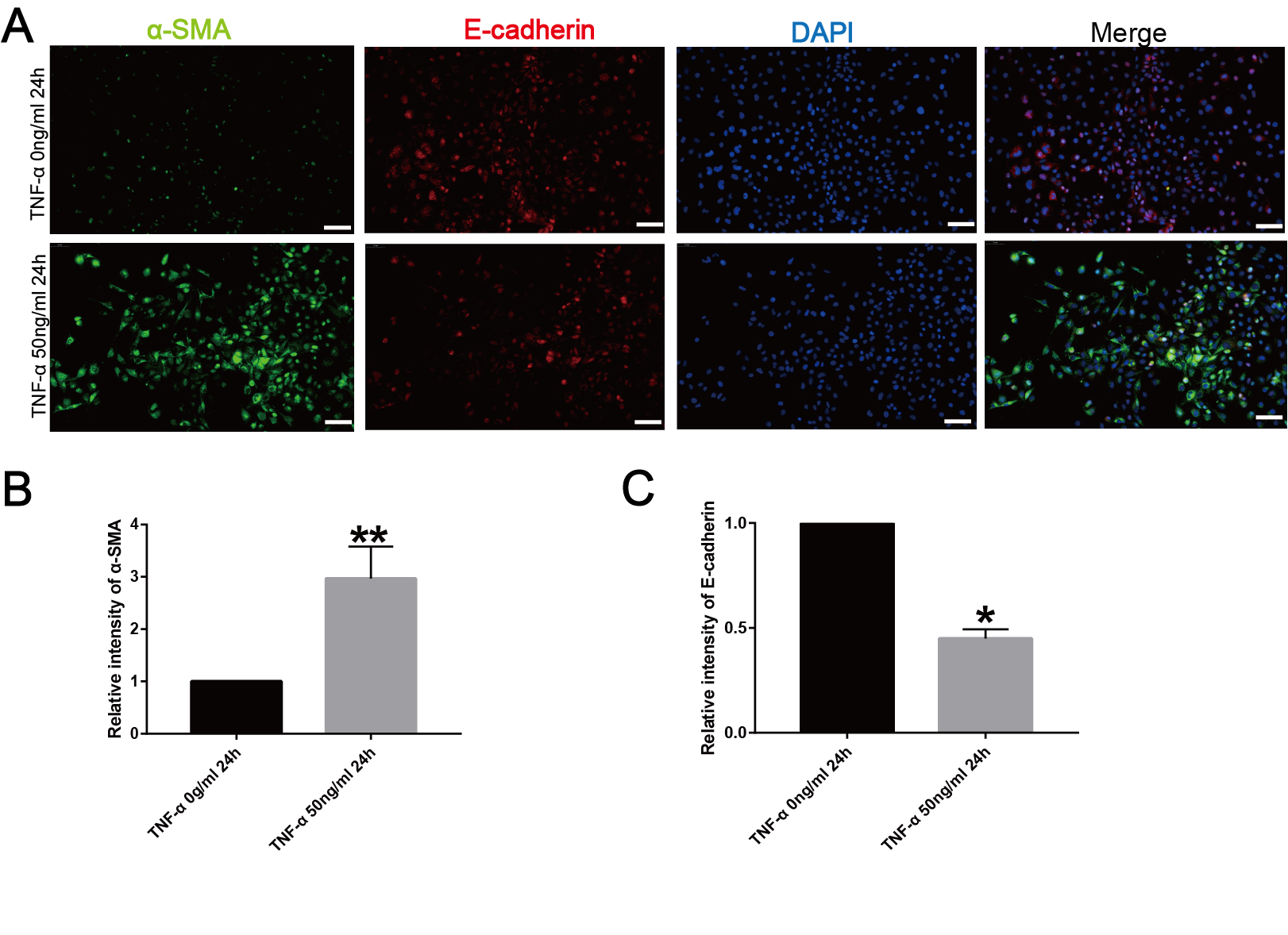

Supplement: Supplementary file 1 [file JCMM-23-5390-s001.tif]

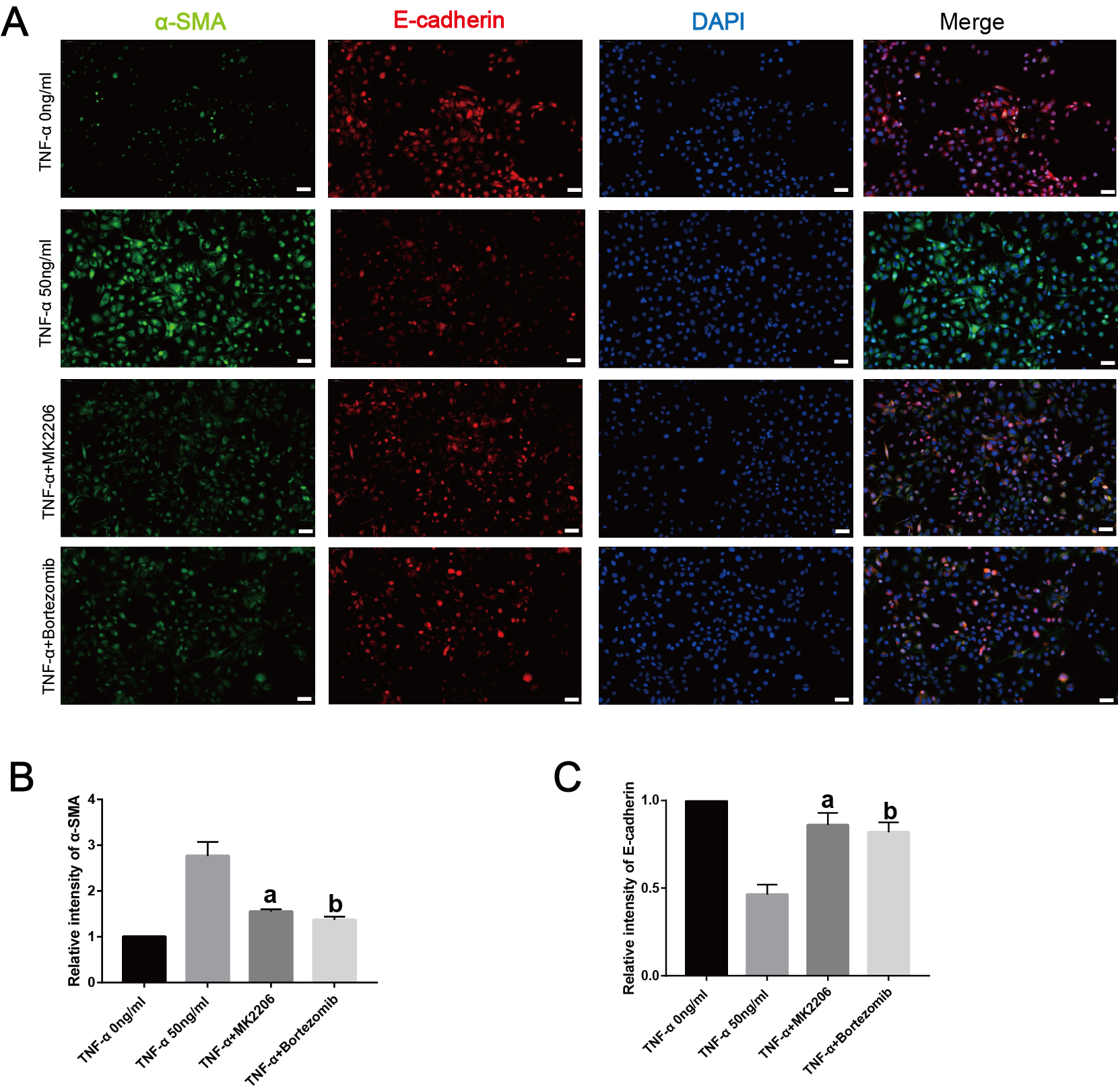

Supplement: Supplementary file 2 [file JCMM-23-5390-s002.tif]

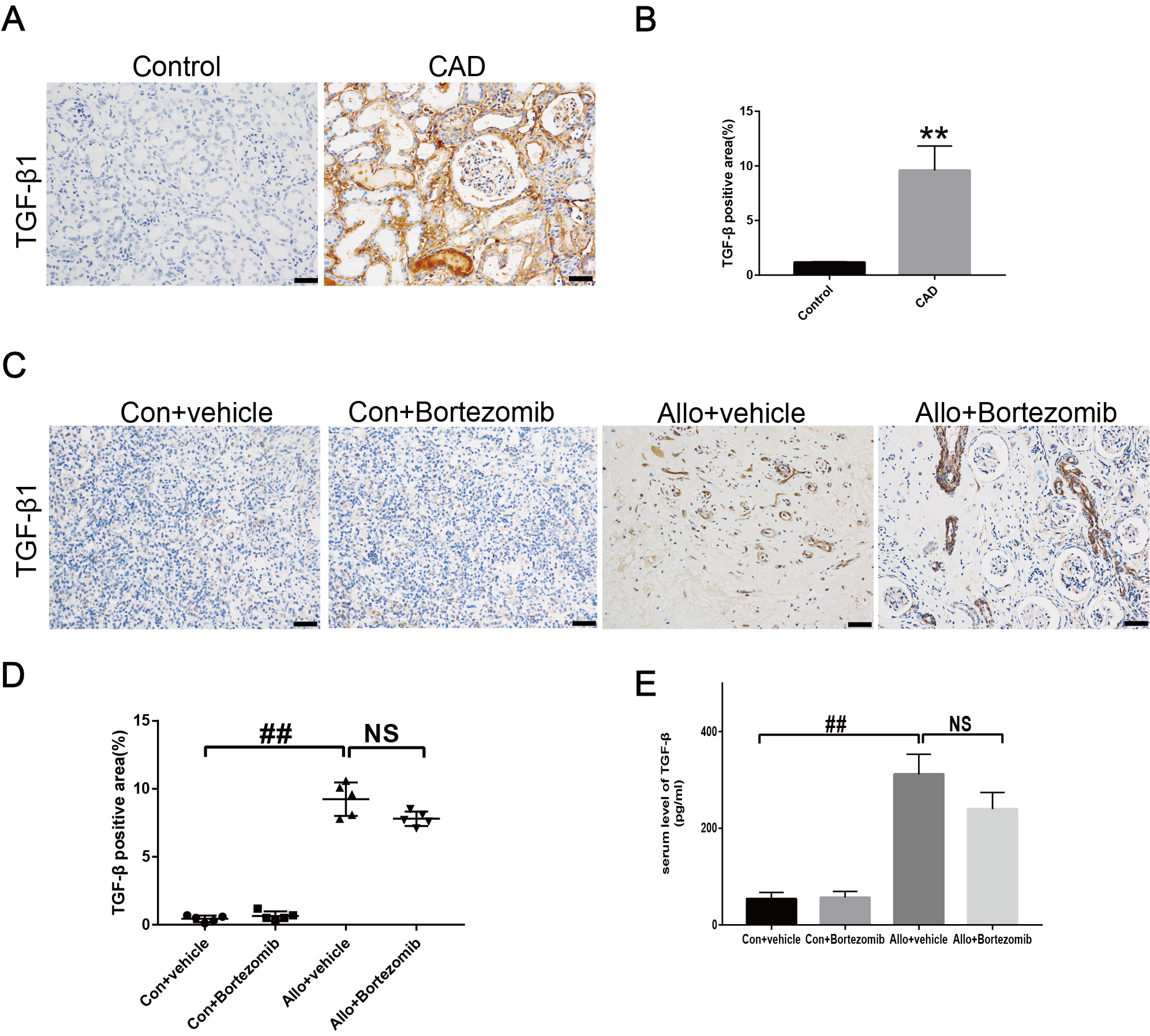

Supplement: Supplementary file 3 [file JCMM-23-5390-s003.tif]

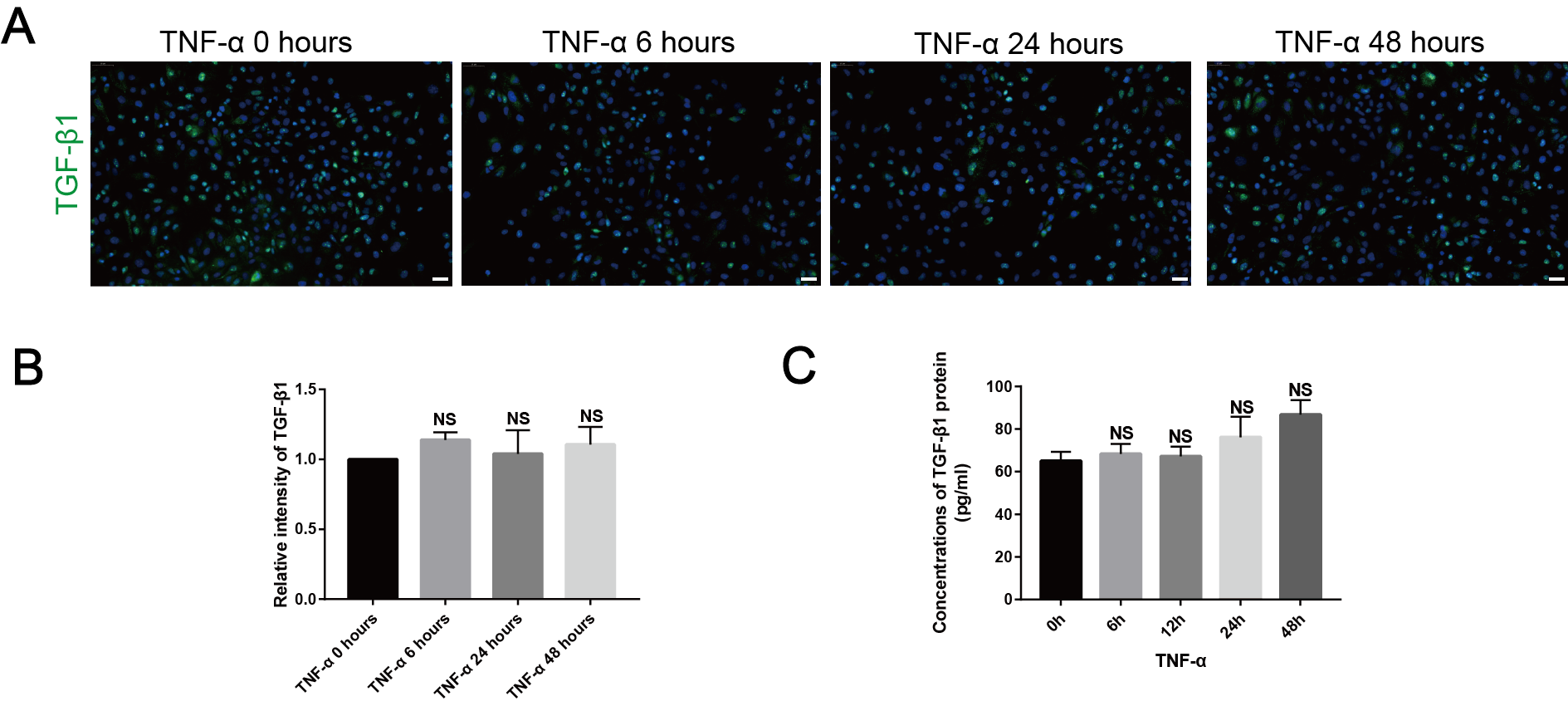

Supplement: Supplementary file 4 [file JCMM-23-5390-s004.tif]

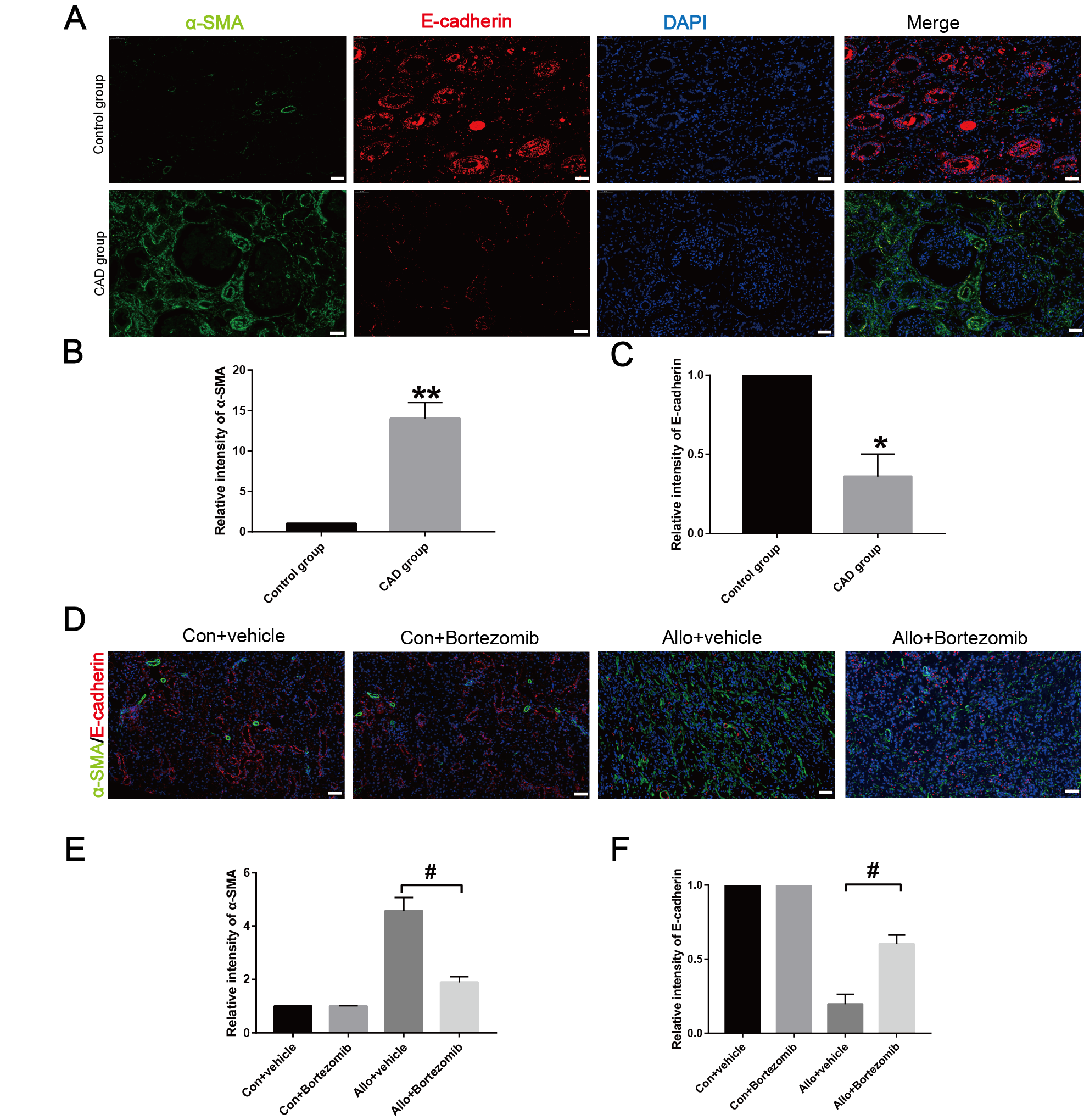

Supplement: Supplementary file 5 [file JCMM-23-5390-s005.tif]
